# Supplementary material for: CryoEM structures of anion exchanger 1 capture multiple states of inward- and outward-facing conformations
Source: Commun Biol. 2022 Dec 14;5:1372. doi: 10.1038/s42003-022-04306-8 (PMC9751308; doi:10.1038/s42003-022-04306-8)
Supplement: Supplementary file 2 — Supplementary information [file 42003_2022_4306_MOESM2_ESM.pdf]

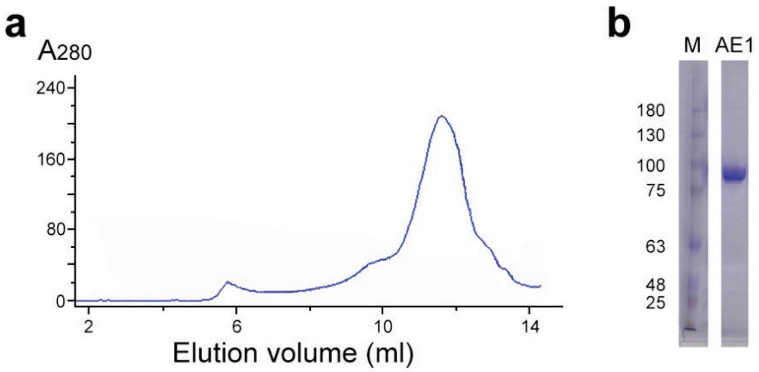

Supplementary Fig. 1 a,b. Biochemical characterization of bovine AE1 purification. a Size-exclusion chromatography of AE1 sample after ion-exchange chromatography. The density large peak corresponding to AE1 dimers, was used for cryoEM studies. **b** SDS-PAGE of the sample used for cryoEM studies.

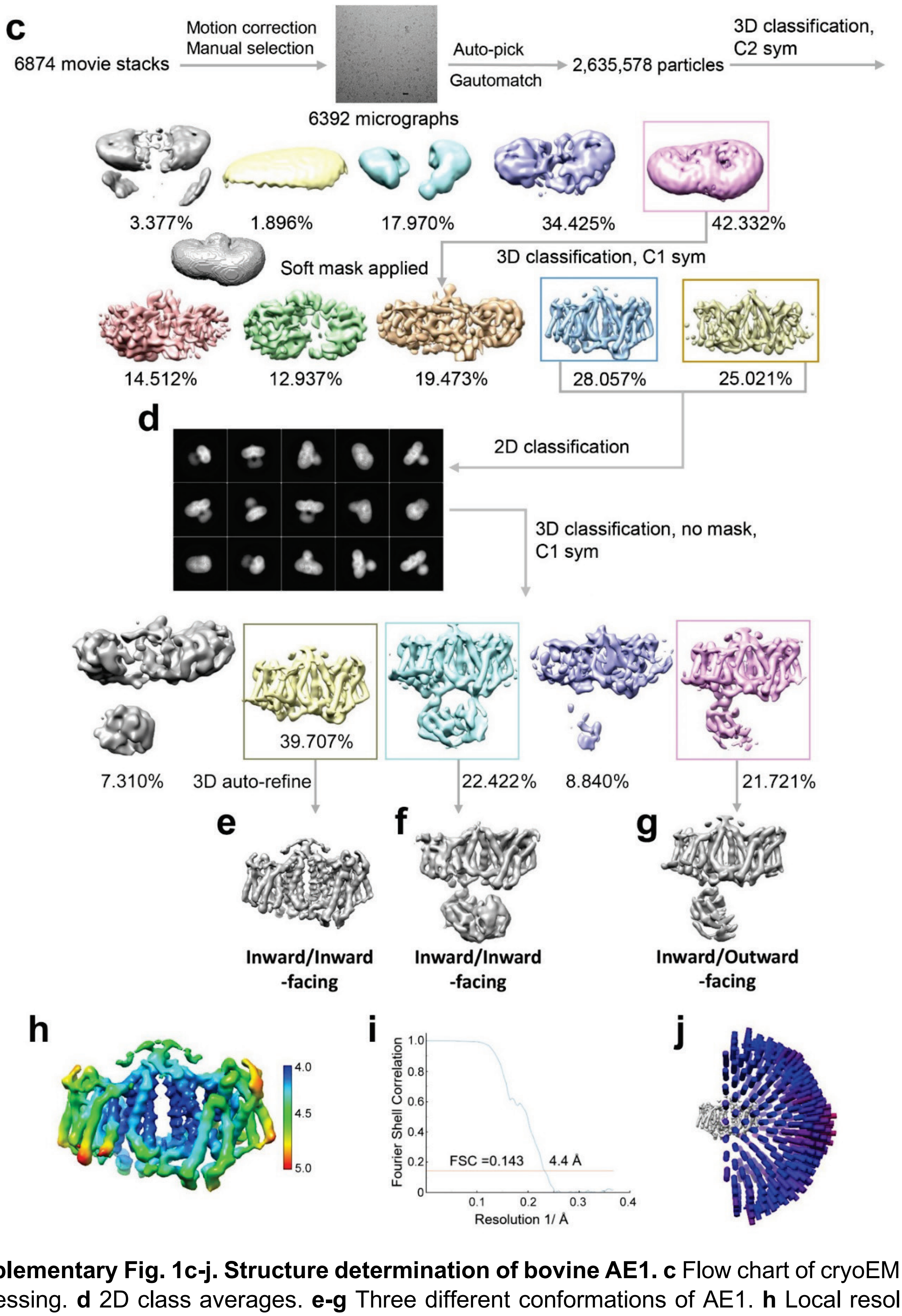

Supplementary Fig. 1c-j. Structure determination of bovine AE1. c Flow chart of cryoEM data processing. **d** 2D class averages. **e-g** Three different conformations of AE1. **h** Local resolution analysis of map **e** by Resmap[1]**. i** FSC curve of the final 3D reconstruction of map **e**. **j** Angular distribution of map **e**.

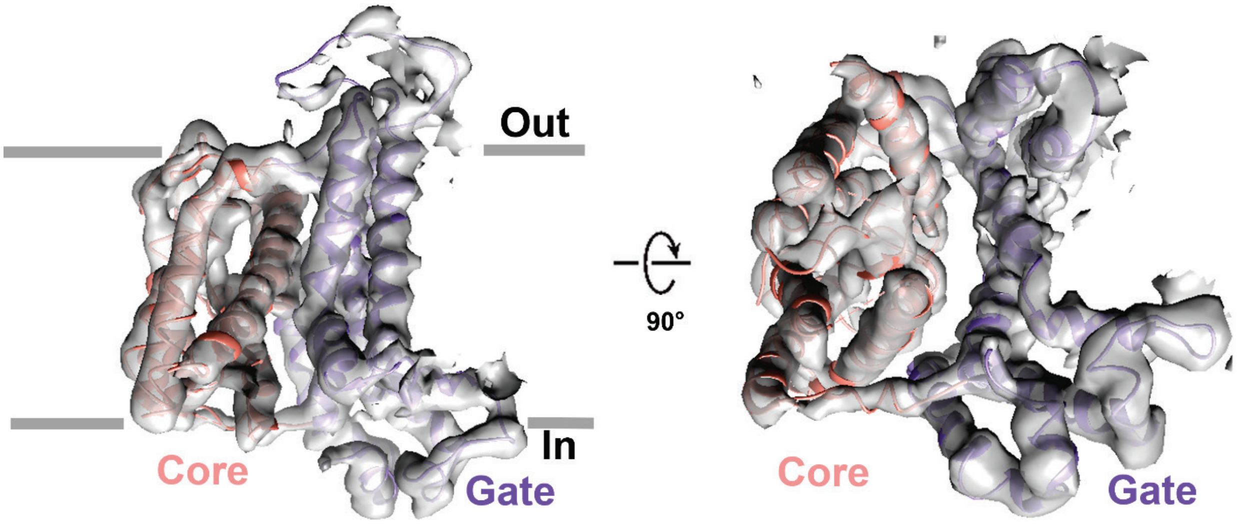

Supplementary Fig. 2. Gate and core domains in bovine AE1. Two perpendicular views of the atomic model in IF state superposed with the cryoEM map. Core domain (salmon) and gate domain (purple).

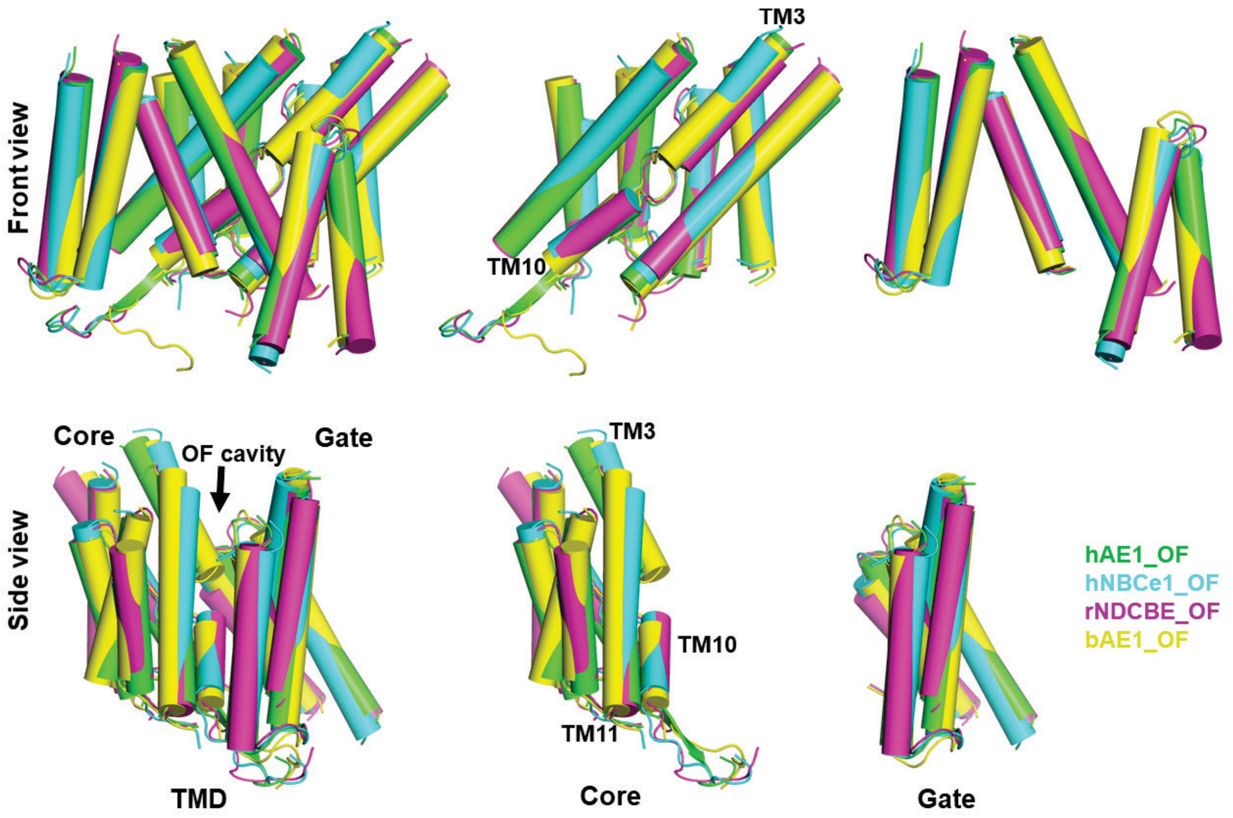

Supplementary Fig. 3. Overlap of the OF states of four available SLC4 structures. Front and side views of human AE1[2] (hAE1, green), human NBCe1[3] (hNBCe1, cyan), rat NDCBE[4] (rNDCBE, magenta), and bovine AE1 (bAE1, yellow) are shown. The core and gate domains are shown separately, for clarity.

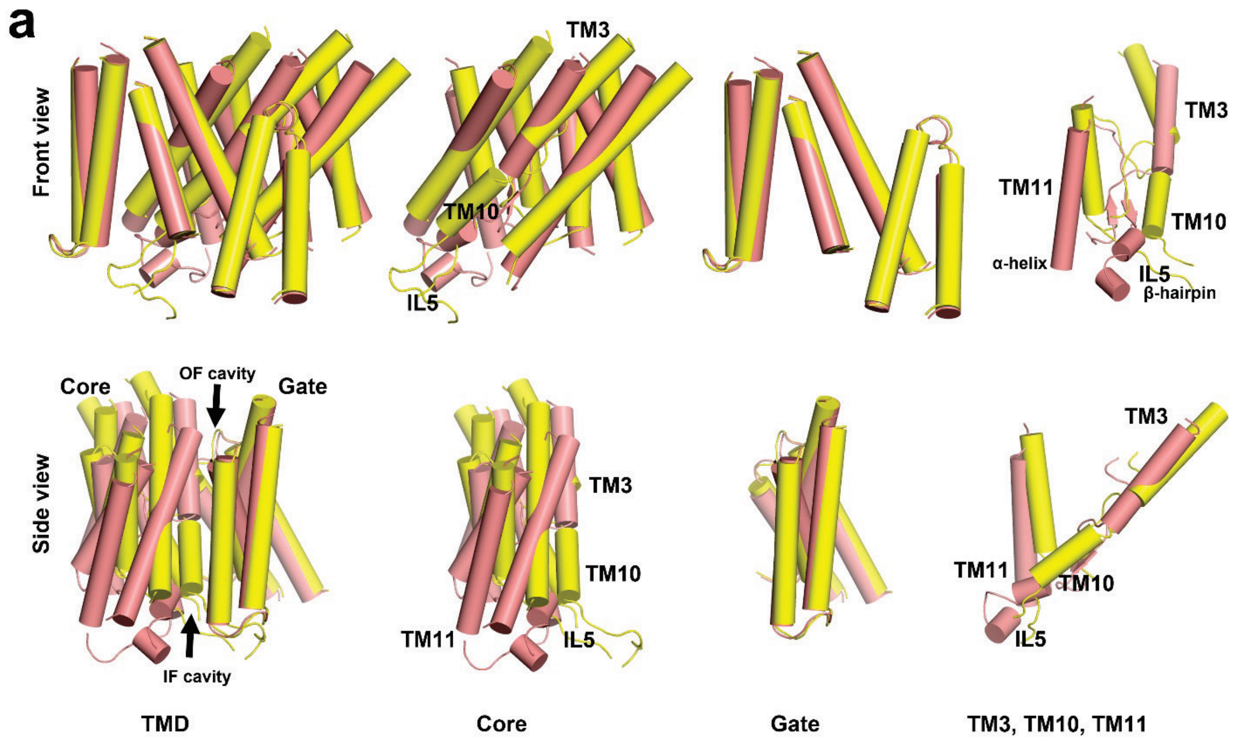

Supplementary Fig. 4a. Comparison of the OF (yellow cylinders) and IF (salmon cylinders) states of AE1. The OF and IF cavities between the core and gate domains are labeled with black arrows. The gate and core domains as well as the pronounced changes in TMs 3, 10, and 11 and the β-hairpin and α-helical portions of IL5 in the OF and IF states, respectively, are shown separately on the right for clarity.

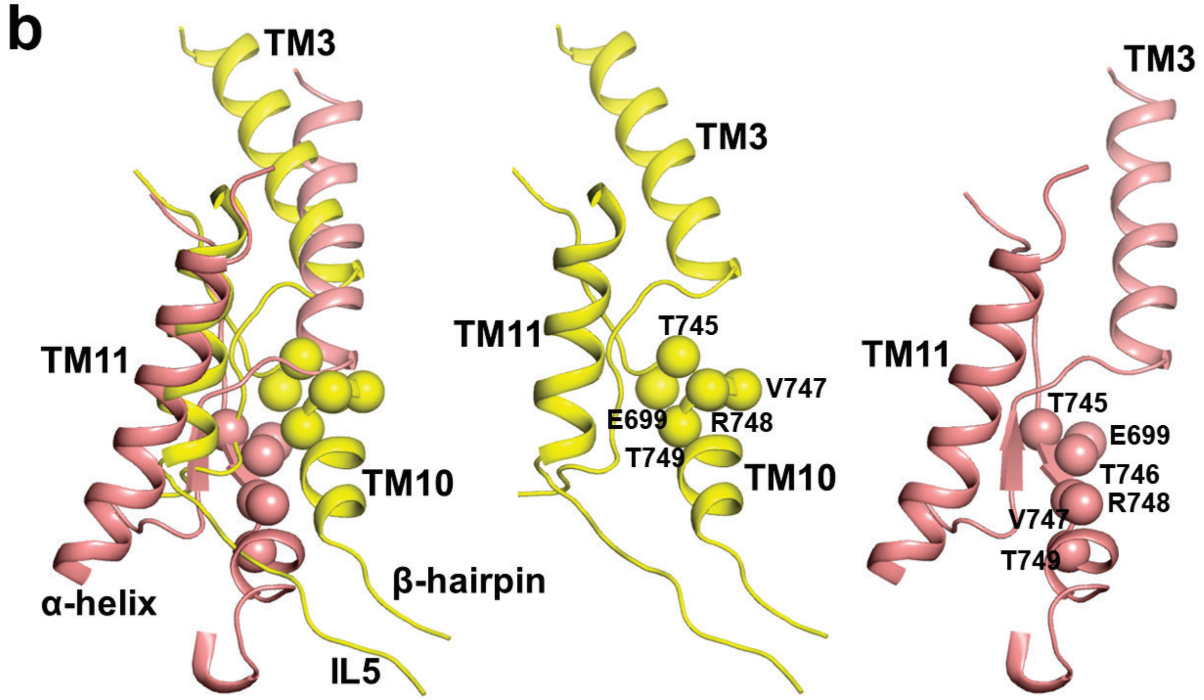

Supplementary Fig. 4b. Architecture of TMs 3, 10 and 11 in bovine OF (yellow) and IF (salmon) conformation illustrating the ~5 Å vertical shift of the ion coordination site between these conformations (evaluated as center of mass shift for the displayed residues)**.** The Cα atoms of the core residues from the central binding site S1[5] are shown as spheres and labeled accordingly.

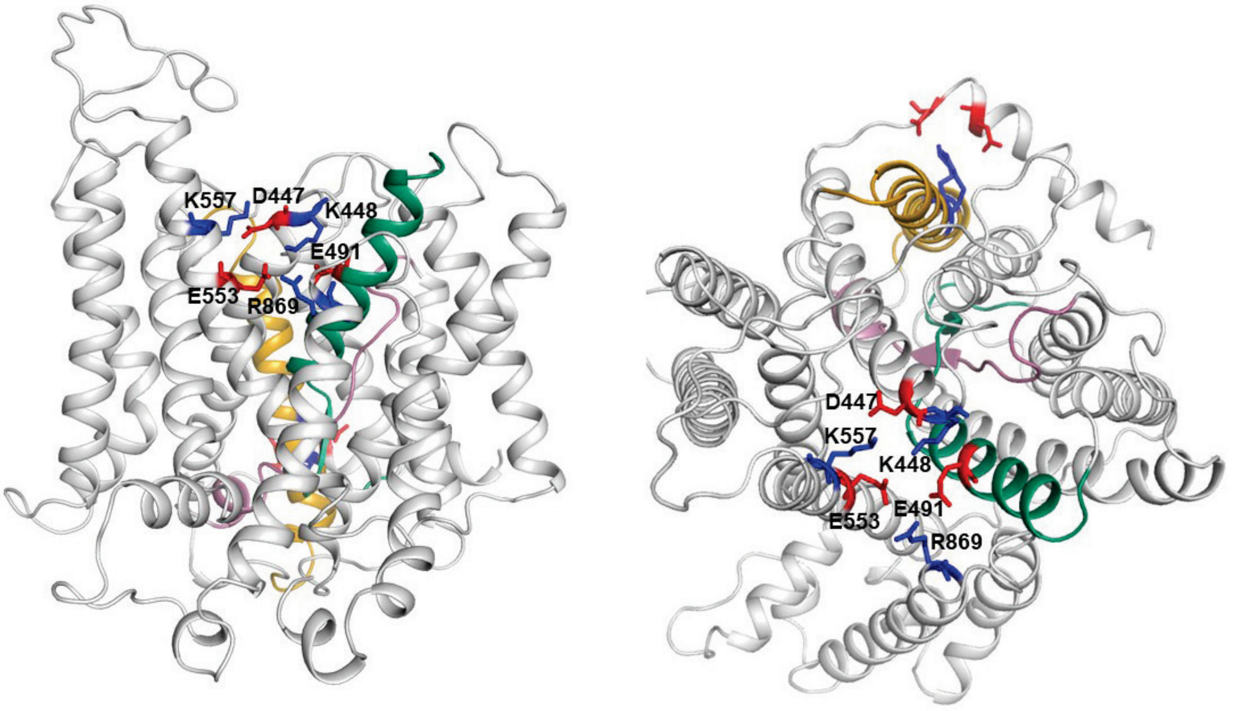

Supplementary Fig. 5. Salt bridges in IF state of bovine AE1. Side and top views. Colors: TM3 (green helix), TM10 (pink helix), TM11 (orange helix), negatively charged residues (red sticks), positively charged residues (blue sticks).

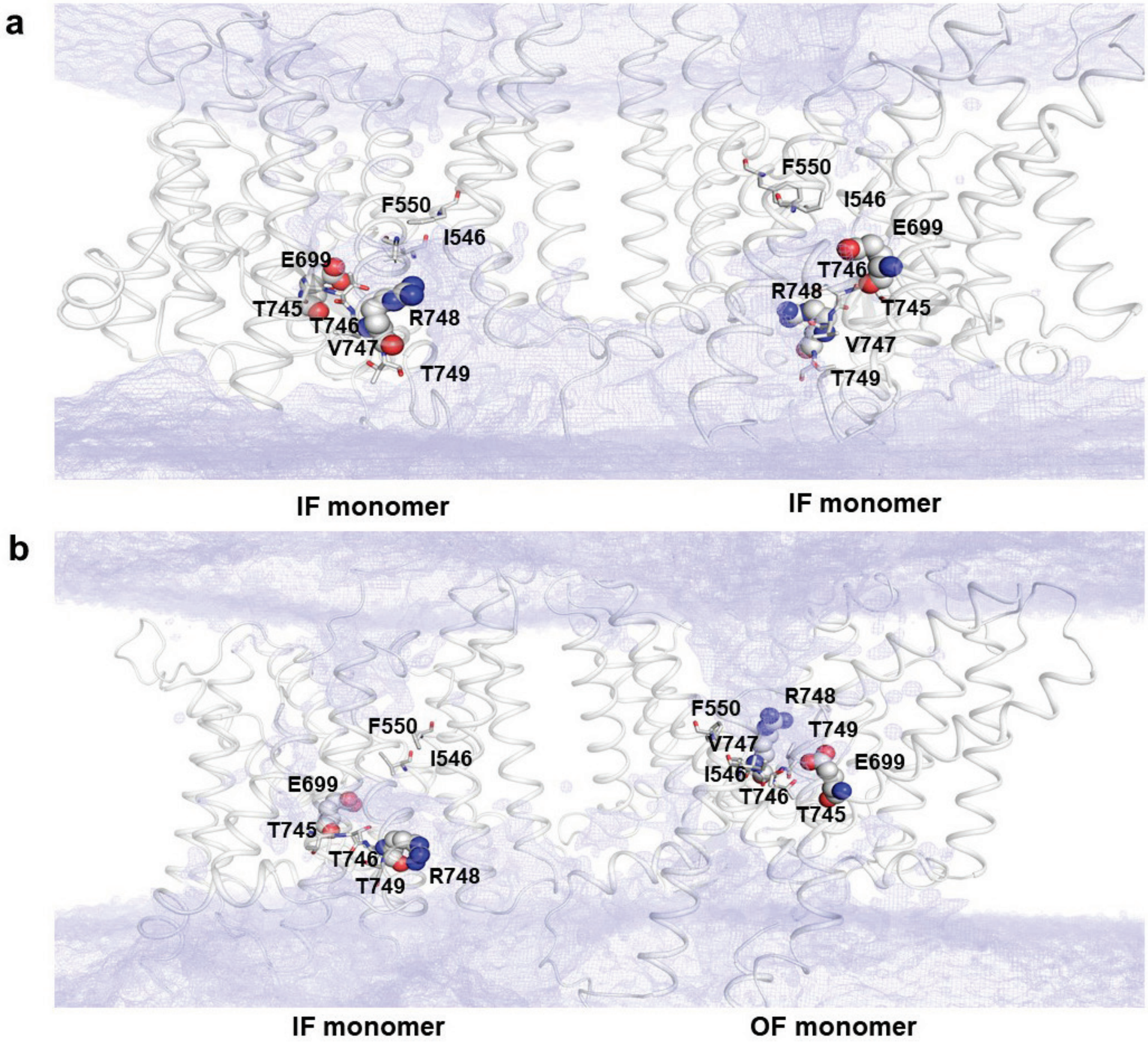

Supplementary Fig. 6. Water maps (blue mesh) averaged from 3 replicas of 1 μs MD trajectories of bovine AE1 (a) IF–IF and (b) IF–OF dimers (contour isovalue 0.5). The residues from the central site S1 in OF state of AE1 are shown as sticks. The two key residues R748 and E699 are shown as spheres for clarity.

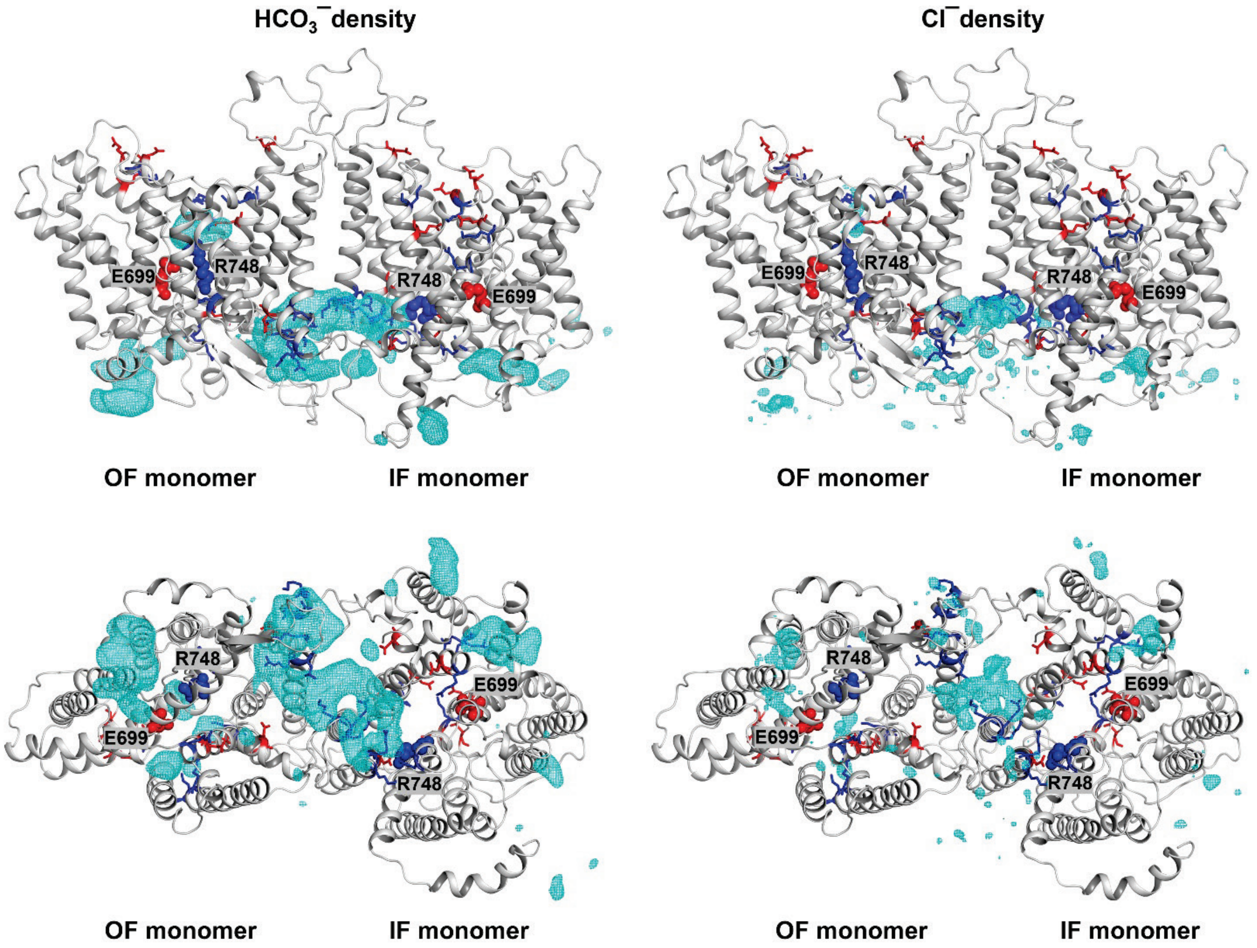

Supplementary Fig. 7. The side and bottom views of HCO_3^- (left) and Cl^- (right) density maps (cyan mesh) of the IF–OF dimer. Data was averaged from three 1 μs MD IF-OF dimer trajectories. Positively and negatively charged residues lining the OF and IF cavities are shown as blue and red sticks, respectively. Residues R748 and E699 from site S1 in OF AE1[5] are shown as blue and red spheres, respectively. Contour isovalues 0.05 (HCO_3^-) and 0.007 (Cl^-).

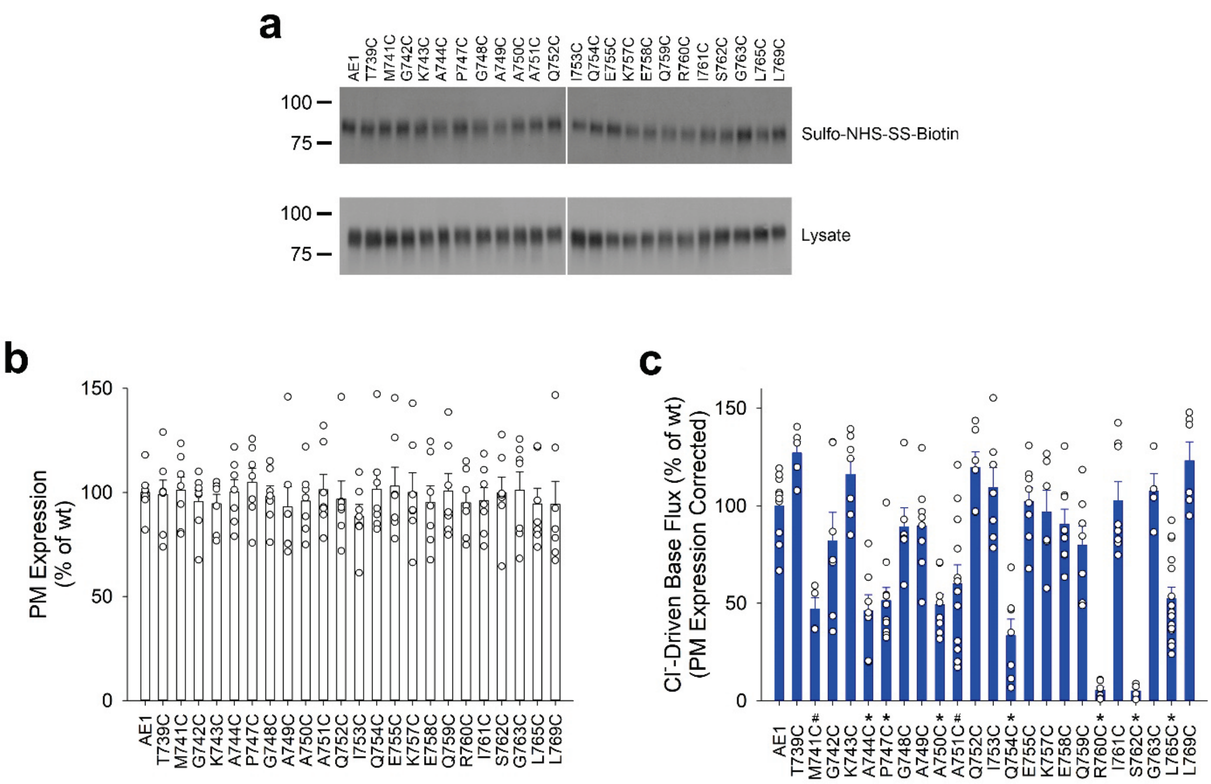

Supplementary Fig. 8. Cell-surface expression and functional analysis of AE1 mutants. a Representative experiments showing immunoblot analysis of cell-surface and cell-lysate expression of wt and mutant AE1 proteins (n = 7 biologically independent experiments). The positions of molecular weight size markers (kDa) are shown on the left. Blot splicing is indicated with a vertical white line. Source data are provided as a Source Data file. **b** Densitometry analysis of cell-surface expression (depicted as percent of wt-AE1). One-way ANOVA was used to compare multiple study group means with wt-AE1. Mutant AE1 data was not statistically different from wt-AE1. Results are depicted as mean ± SEM (n = 7 biologically independent experiments). Open circles represent individual data points. Source data are provided as a Source Data file. **c** AE1 Cl^--driven base transport function was corrected for cell-surface plasma membrane (PM) expression. The data is depicted as percent of wt-AE1 function divided by percent of wt-AE1 cell-surface PM expression. AE1 wt (n = 10 biologically independent experiments) and single cysteine functional mutant data: T739C (n = 5, p = 0.3777); M741C (n = 4, p = 0.0037); G742C (n = 7, p = 0.7377); K743C (n = 7, p = 0.8297); A744C (n = 7, p = 0.0001); P747C (n = 10, p = 0.0002); G748C (n = 6, p = 0.9798); A749C (n = 8, p = 0.9735); A750C (n = 8, p = 0.0002); A751C (n = 12, p = 0.0020); Q752C (n = 6, p = 0.6946); I753C (n = 7, p = 0.9877); Q754C (n = 7, p < 0.0001); E755C (n = 8, p = 1.0000); K757C (n = 6, p = 1.0000); E758C (n = 8, p = 0.9841); Q759C (n = 7, p = 0.6158); R760C (n = 6, p < 0.0001); I761C (n = 8, p = 1.0000); S762C (n = 4, p < 0.0001); G763C (n = 4, p = 0.9988); L765C (n = 15, p < 0.0001); and L769C (n = 6, p = 0.5080). One-way ANOVA and Dunnett's test were used to compare multiple study group means with wt-AE1. Statistically significant results differing from wt-AE1 are depicted as mean ± SEM (#p < 0.005 and *p < 0.001). Source data are provided as a Source Data file.

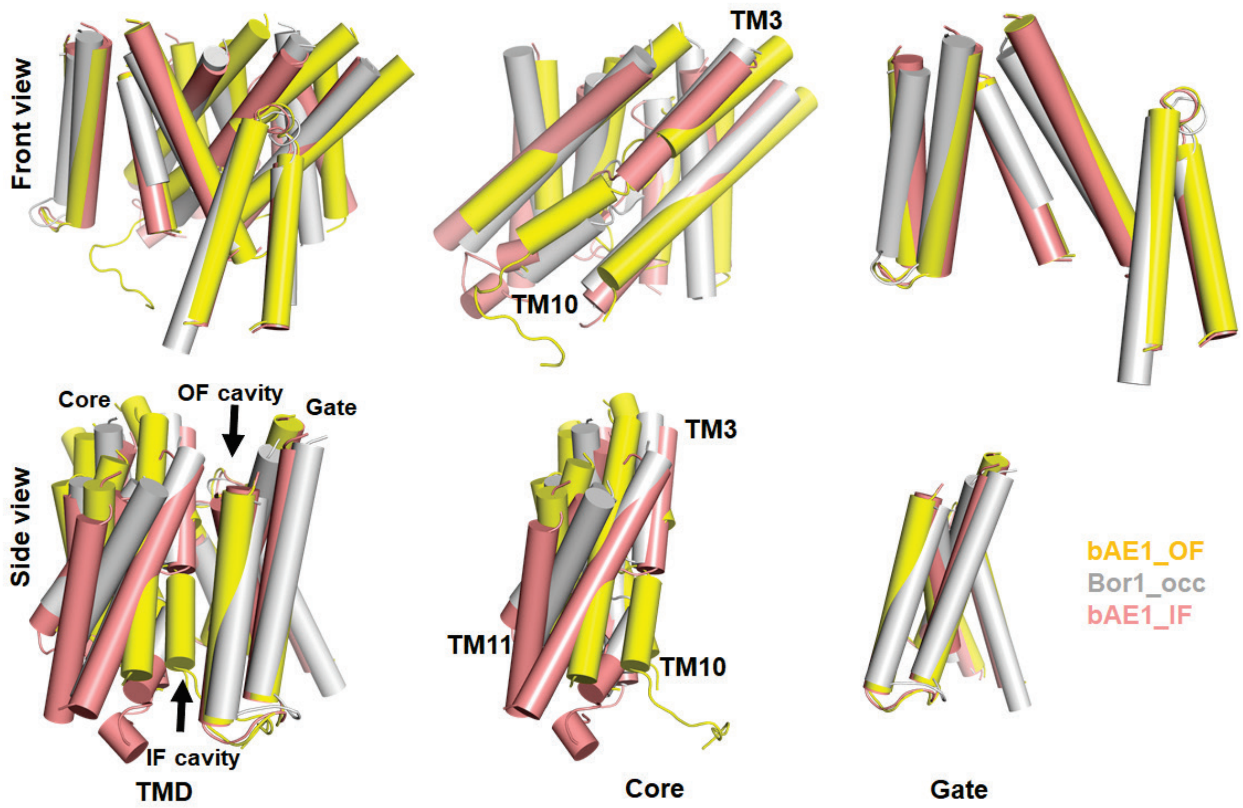

Supplementary Fig. 9. Comparison of the OF and IF bovine AE1 (bAE1) states (yellow and salmon cylinders, respectively) and the occluded Bor1 structure[6] (grey cylinders) illustrating the elevator transport mechanism of AE1. Positions of the OF and IF cavities between the gate and core domains are shown with black arrows.

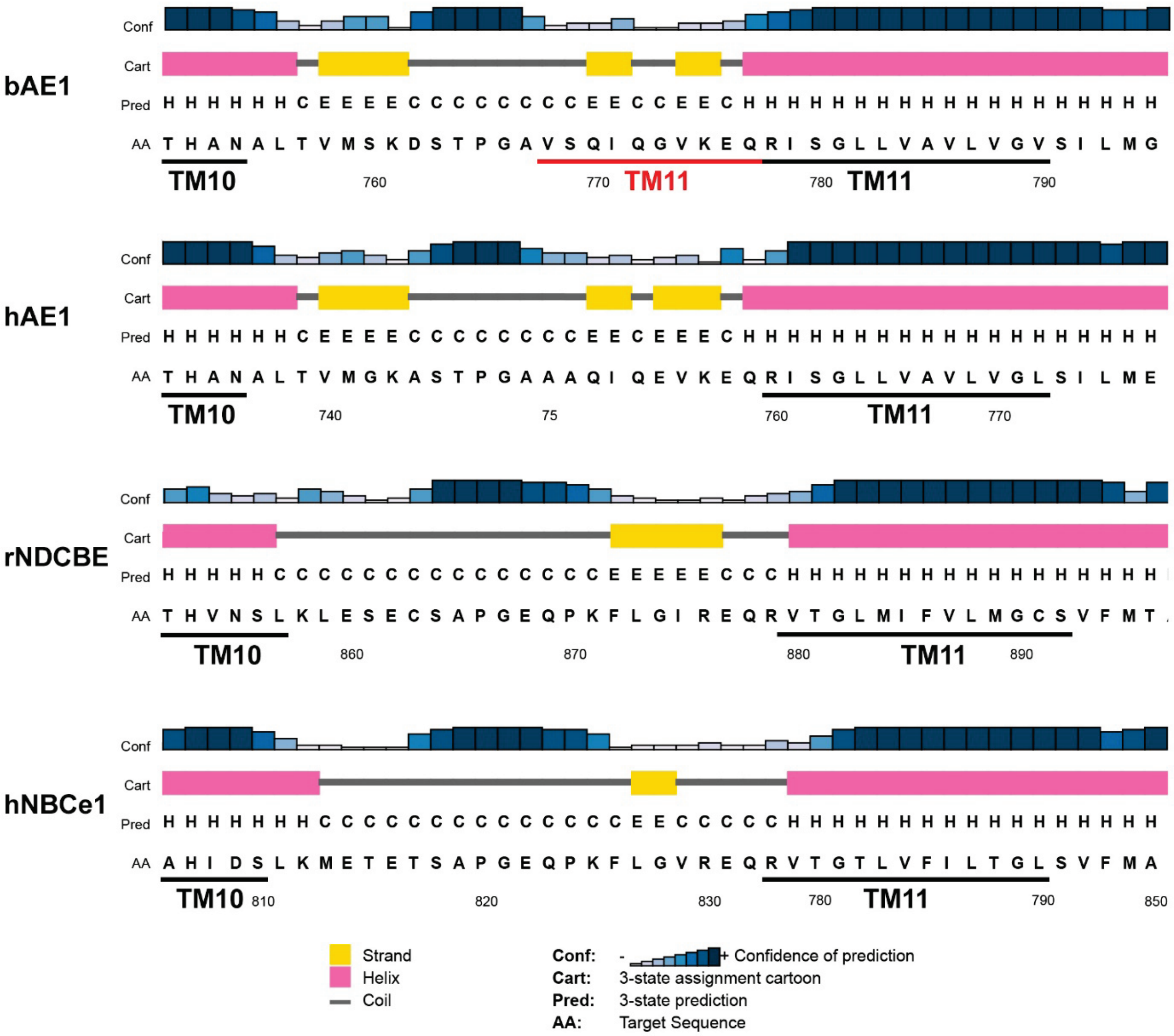

Supplementary Fig. 10. Secondary structure evaluation in the TM10-IL5-TM6 area in AE1 and the SLC4 transporters with known atomic structures using PSIPRED analysis[7]. The positions of TM10 and TM11 in the OF conformation of bovine AE1 (bAE1), human AE1 (hAE1)[2], rat NDCBE (rNDCBE)[4] and human NBCe1 (hNBCe1)[3] are shown with black line. The IL5 sequence included in TM11 in the IF conformation of bovine AE1 (bAE1, present study) is shown with red line.

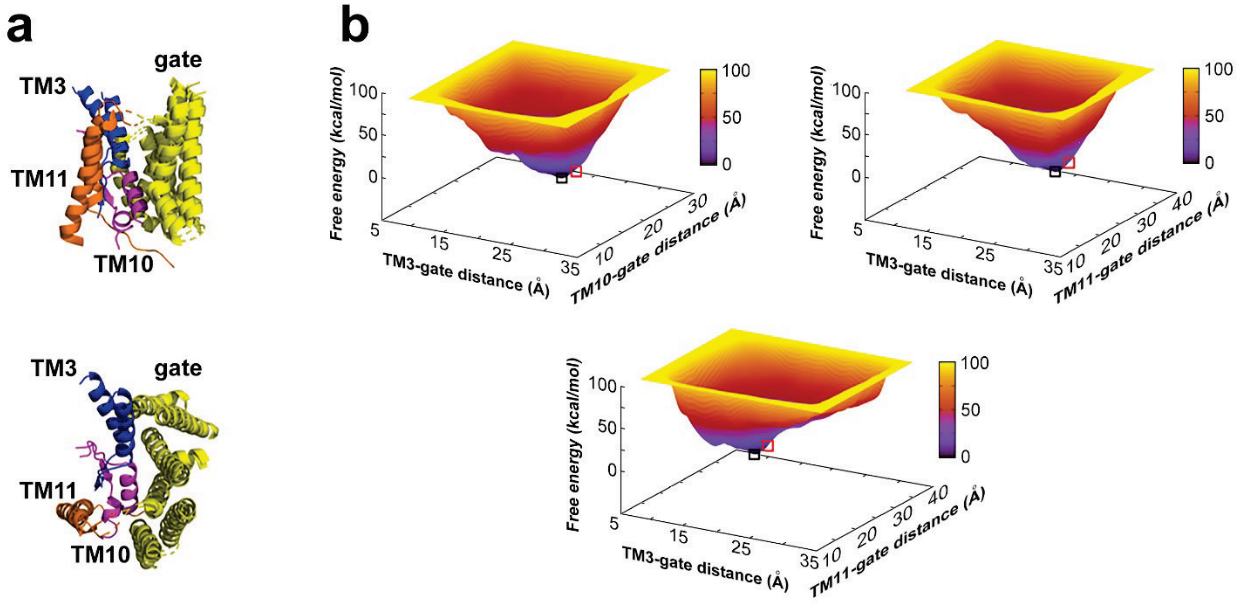

Supplementary Fig. 11: Free energy surfaces of bovine AE1 calculated using metadynamics simulations. Three collective variables (CVs) were used: distance between centres of mass of the TM3, TM10, TM11 helix and the immobile gate part. **a** Protein segments used for the collective variable definitions. IF and OF states are superimposed. Gate (yellow) part is immobile whereas TM3 (blue), TM10 (magenta), and TM11 (orange) change their relative position with respect to the gate region. Upper panel presents superimposed side view, lower panel presents top view. **b** Free energy surface projected along two CVs at a time. The free energy landscape highlights OF state (black square) to be the global minima and IF state (red square) is separated by at least 5 kcal/mol. The coarse-grained metadynamics methodology is described in Supplementary Methods (see below).

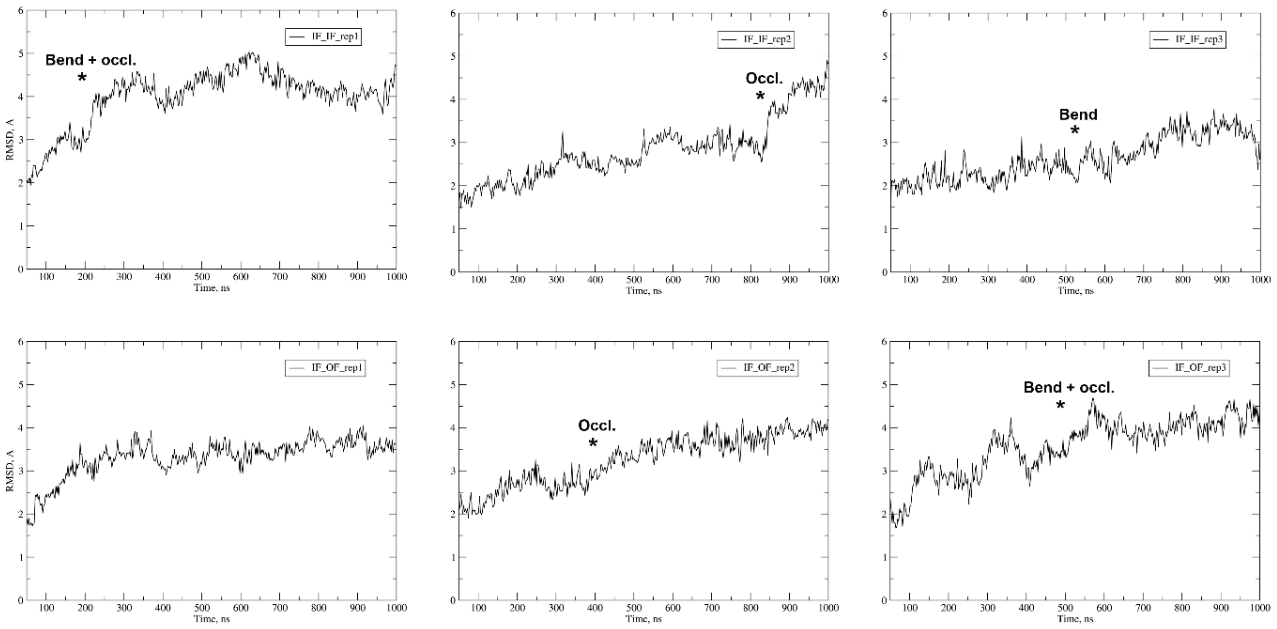

Supplementary Fig. 12. RMSD plots for the 1 μs MD simulations of bAE1 dimers. Relevant structural changes during the simulations (i.e. bending of TM11 and occlusion of the IF cavity by motion of TM10) that impact the RMSD are marked with an asterisk. The first 50 ns of the trajectories were discarded from the RMSD analysis.

Supplementary Table S1

Simulation details and system composition of the IF-IF and IF-OF replicas used for 1 μs MD simulations

	IF-IF dimer	IF-OF dimer
Number of replicas	3	3
Production time (per replica)	1 μs	1 μs
Box dimensions	175.8 x 175.8 x 111.9 Å	175.5 x 175.5 x 115.6 Å
Box type	Rectangular	Rectangular
Total number of atoms	321,885	331,988
Number of water molecules	66,735	70,092
Salt concentration	0.075M NaCl + 0.075 M $NaHCO_3$	0.075M NaCl + 0.075 M $NaHCO_3$
Lipid type	POPC	POPC
Number of lipids (upper leaflet)	394	394
Number of lipids (lower leaflet)	384	384

Supplementary Methods

Coarse-Grained Metadynamics Simulations

We employed coarse-grained metadynamics simulations to probe the free energy landscape of the IF to OF conformational transition and to obtain a qualitative understanding about which state is more energetically favorable. Three collective variables (CVs) were employed for the description of the IF-OF transition. The CVs were selected using the immobile gate (TM 5-7, 12-14) part as the reference. The center of mass (COM) distances of the core helices (TM 1-4, 8-11) from the COM of the gate was calculated for both the IF and OF states. The COM distances of these helices from the core part were used as CVs if they varied more than 2 Å, which led to three CVs, namely COM distances between TM3-gate, TM10-gate and TM11-gate.

The simulations were performed with the Martini 3.0 force field[8] and the go-model for description of the protein as implemented in Martini 3.0. The OF monomer was used as the starting point of the OF to IF transition and was embedded in a POPC bilayer of 315 lipids (160 and 155 in upper and lower leaflets, respectively). The protein and lipid were further solvated with 6,567 water molecules and 0.1 M NaCl. Afterwards, the system was subjected to energy minimization for 500 steps using the steepest-descent algorithm, followed by 1 ns equilibration in isothermal-isobaric (NPT) ensemble with a timestep of 20 fs and the leap-frog integrator. Finally, we performed the metadynamics calculations for 10 µs in NPT ensembles using the same timestep and integrator. The system pressure and temperature were set to 1 bar and 310 K, respectively; the pressure was maintained using Parrinello-Rahman barostat and the temperature was maintained using v-rescale thermostat. A semi-isotropic pressure coupling was used to maintain the shape of the bilayer where bilayer lateral dimensions were coupled. Pair list was generated using the Verlet scheme for the nonbonded interactions. The coulombic terms were calculated using reaction-field electrostatics with a cut-off of 1.1 nm and the relative dielectric constant was set to 15. A cutoff value of 1.1 nm was used for the VdW terms using a potential-shift with Verlet cutoff-scheme.

All the calculations were carried out with GROMACS (v. 2019.6)[9] and PLUMED (v. 2.7.0)[10]. The metadynamics calculations made use of the three collective variables mentioned above. The height of the gaussian was chosen as 0.5 kJ/mol, and sigma (width) was set to 0.05. The gaussians were deposited every 500 timesteps.

Due to the coarse-grained nature of the metadynamics protocol, the complexity of the assessed conformational transition, the lack of bound substrates, and the presence of only one

monomer in the simulation, the intrinsic convergence of the metadynamics results is not suitable for assessment of their accuracy. Thus, our coarse-grained metadynamics results should be viewed from a qualitative (e.g. which state is of lower energy) rather than a quantitative (exact energy differences) point of view. All-atom metadynamics simulations which address the deficiencies mentioned above and can provide quantitatively accurate energetics for the IF to OF transition are currently underway.

Supplementary References

1. Kucukelbir, A., Sigworth, F.J. & Tagare, H.D. Quantifying the local resolution of cryo-EM density maps. *Nat. Methods* **11**, 63–65 (2014).
2. Arakawa, T. et al. Crystal structure of the anion exchanger domain of human erythrocyte band 3. *Science* **350**, 680–684 (2015).
3. Huynh, K.W. et al. CryoEM structure of the human SLC4A4 sodium-coupled acid-base transporter NBCe1. *Nat. Commun.* **9**, 900 (2018).
4. Wang, W. et al. Cryo-EM structure of the sodium-driven chloride/bicarbonate exchanger NDCBE. *Nat. Commun.* **12**: 5690 (2021).
5. Zhekova, H.R., Pushkin, A., Kayık, G., Kao, L., Azimov, R., Abuladze, N., Kurtz, D., Damergi, M., Noskov, S.Y. & Kurtz, I. Identification of multiple substrate binding sites in SLC4 transporters in the outward-facing conformation: Insights into the transport mechanism. *J. Biol. Chem.* **296**, 100724 (2021).
6. Thurtle-Schmidt, B.H. & Stroud, R.M. Structure of Bor1 supports an elevator transport mechanism for SLC4 anion exchangers. *Proc. Natl. Acad. Sci. USA* **113**, 10542–10546 (2016).
7. McGuffin, L.J., Bryson, K. & Jones, D.T. The PSIPRED protein structure prediction server. Bioinformatics 16, 404–405 (2000).
8. Souza, P.C.T. et al. Martini 3: a general purpose force field for coarse-grained molecular dynamics. *Nature Methods* **18**, 382–388 (2021).
9. Pronk, S. et al. GROMACS 4.5: a high-throughput and highly parallel open source molecular simulation toolkit. *Bioinformatics* **29**, 845–854 (2013).
10. Tribello, G.A., Bonomi, M., Branduardi, D., Camilloni, C. & Bussi, G. PLUMED 2: New feathers for an old bird. *Comp. Physics Commun.* **185**, 604–613 (2014).
