# Supplementary material for: CryoEM structures of anion exchanger 1 capture multiple states of inward- and outward-facing conformations
Source: Commun Biol. 2022 Dec 14;5:1372. doi: 10.1038/s42003-022-04306-8 (PMC9751308; doi:10.1038/s42003-022-04306-8)
Supplement: Supplementary file 3 — Description of Additional Supplementary Files [file 42003_2022_4306_MOESM3_ESM.docx]

**File Name: Supplementary Movie 1**

**Description:** Rigid core motion (core rotation and downward core shift) representing the elevator-like OF to IF transformation of the core in bovine AE1. The overall downward core displacement can be regarded as a vertical shift (along the z axis) of less than 5 Å, combined with a small lateral movement in the xy plane. The motion involves predominantly the core, while the gate reorganizes minimally (RMSD ~ 0.99 Å for gate in IF and OF state). The downward shift of the core leads to a downward slide of the core binding site residues (indicated as green, red, and yellow spheres) of ~5 Å. The colors of the highlighted helices are as follows: blue (core domain), purple (gate domain).

**File Name: Supplementary Movie 2**

**Description:** Side view of the OF to IF transition of a bovine AE1 monomer simulated with eBDIMS, demonstrating the elevator-like vertical shift of the core with respect to the gate and the folding/unfolding of TM11. The colors of the highlighted helices are as follows: blue (gate domain), green (TM3), magenta (TM10), orange (TM11).

**File Name: Supplementary Movie 3**

**Description:** Bottom view of the OF to IF transition of a bovine AE1 monomer simulated with eBDIMS, demonstrating the folding/unfolding of TM11 during the transport cycle. The colors of the highlighted helices are as follows: blue (gate domain), green (TM3), magenta (TM10), orange (TM11).

**File Name: Supplementary Movie 4**

**Description:** Bottom view of a mixed IF–OF dimer, demonstrating occlusion of the IF cavity of the IF monomer during a representative MD simulation. The colors of the highlighted helices are as follows: green (TM3), magenta (TM10), orange (TM11).

**File Name: Supplementary Movie 5**

**Description:** Side view of an IF dimer, demonstrating bending in TM11 during the concerted motion of H1 and TM11 and occlusive motion of TM10 within the IF cavity. The colors of the highlighted helices are as follows: green (TM3), magenta (TM10), orange (TM11).

**File Name: Supplementary Data 1**

**Description:** Initial and final steps from the MD trajectories and sample input files for Anton2 MD simulations are provided.

**File Name: Supplementary Data 2**

**Description:** Source data files for Fig. 5c**,** Supplementary Fig. 1b, Supplementary Fig. 8a and 8b
